# The complete chloroplast genome of the medicinal plant *Polygonum cuspidatum* (Polygonaceae) and its phylogenetic implications within the subfamily Polygonoideae

**DOI:** 10.1080/23802359.2021.1917313

**Published:** 2021-04-26

**Authors:** Xiaofeng Ye, Jiuguo Lin, Manjia Zhou, Xiangyu He, Meixiu Yan, Rubin Cheng

**Affiliations:** College of Pharmaceutical Science, Zhejiang Chinese Medical University, Hangzhou, China

**Keywords:** Complete chloroplast genome, *Polygonum cuspidatum*, phylogenetic analysis, simple sequence repeats

## Abstract

*Polygonum cuspidatum* Siebold & Zucc. is a well-known and widely used medical plant to treat arthritis, gout and inflammation. In this study, we determined the complete chloroplast genome sequence of *P. cuspidatum* from Zhejiang Province. The assembled chloroplast (cp) genome was 163,183 bp in length, containing two inverted repeated (IR) regions of 30,859 bp each, a large single copy (LSC) region of 87,905 bp, and a small single copy (SSC) region of 13,560 bp. The genome encodes 131 genes, consisting of 86 protein-coding, 37 tRNA, and eight rRNA genes. The overall GC content of *P. cuspidatum* is 37.53%, with the highest GC content of 41.27% in the IR region. The 86 protein-coding genes encode 27,597 amino acids in total, most of which use the initiation codon ATG, except the *ndhD* gene which starts with ACG. The length of the tRNA genes range from 48 bp to 88 bp, with the highest GC content of 62.16% in tRNA-Arg (ACG) and tRNA-Asp (GUC). A total of 66 simple sequence repeats are identified in the cp of *P. cuspidatum.* Phylogenetic analysis indicated a sister relationship between *P. cuspidatum* and *Fallopia sachalinensis*, suggesting a close genetic relationship between the genera of *Polygonum* and *Fallopia*. This work provides basic genetic resources for investigating the evolutionary status and population genetics of this important medicinal species.

*Polygonum cuspidatum* Siebold & Zucc., commonly name Japanese knotweed, is a large and well-known East Asian perennial plant classified in the family Polygonaceae. Its stem and root have been used as main ingredient of functional foodstuff products in Japan and South Korea (Kirino et al. 2012). Furthermore, *P. cuspidatum* is a traditional and popular medicinal herb in China with therapeutic effects to treat diabetes, cancer, heart disease and other human pathologies (Peng et al. 2013). Stilbenes and quinones are the main active compounds from *P. cuspidatum*, which have exhibit significant anti-inflammatory, antioxidant, and hepatoprotective activities (Huang et al. 2008). This plant is widely cultivated in many provinces of China to produce materials for the extraction of biologically active compound resveratrol, which has been widely utilized in the nutraceutical and cosmetics industries (Mei et al. 2015). However, due to their similar appearance, it is difficult to distinguish the dry roots of *P. cuspidatum* from those of closely related species, such as *Fallopia multiflora* (Thunb.) Haraldson and *Rheum palmatum* L. In addition, the lack of genomic information represents a major obstacle to evaluating the quality and cultivating high quality cultivars of *P. cuspidatum* from different regions of Asia. The medicinal and economic value of *P. cuspidatum* makes its genetic and phylogenetic investigation necessary. Here, we report the complete chloroplast (cp) genome of *P. cuspidatum* from Zhejiang Province, China, to contribute to the development of identification strategies and understanding the genetic diversity and evolutionary history within the subfamily Polygonoideae.

The sample of *Polygonum cuspidatum* was collected from Fuyang area of Zhejiang Province (30°05′2.4″N, 119°53′20.4″E). The specimen was deposited in Medicinal Herbarium Center of Zhejiang Chinese Medical University (Herbarium Code: MHCZCMU; collector: Rubin Cheng, biothcheng@hotmail.com; voucher number: HZZJ-191208; https://yxy.zcmu.edu.cn). Total genomic DNA was extracted and sequenced using the Illumina Hiseq Platform 2500 according to the previous report (Dong et al. 2020; Gao et al. 2020). The raw reads were trimmed using Trimmomatic to remove the low quality reads and adapters. The clean reads were mapped to the homologous species of *Fagopyrum luojishanense* (NC_037706) to obtain the order of contigs (Wang et al. 2018). The matched paired-end reads were assembled by metaSPAdes 3.13.0 to construct the cp genome of *P. cuspidatum* (Nurk et al. 2017). The chloroplast was annotated using GeSeq (https://chlorobox.mpimp-golm.mpg.de/geseq.html) and the primary annotated results were manually verified by BLAST (Tillich et al. 2017). The complete cp genome of *P. cuspidatum* was submitted to GenBank with the accession number of MW411186.

The length of the complete chloroplast (cp) genome of *P. cuspidatum* was 163,183 bp, with a large single copy (LSC) region of 87,905 bp, a small single copy (SSC) region of 13,560 bp, and two separated inverted repeated (IR) regions of 30,859 bp each. A total of 131 genes were identified in the cp of *P. cuspidatum*. The overall base composition was estimated to be 31.07% for A, 19.10% for C, 18.43% for G and 31.40% for T, respectively. The cp of *P. cuspidatum* contained 18 duplicated genes in the IR region, including seven tRNAs, four rRNAs and seven protein-coding genes. The length of the protein coding sequence was 82,718 bp, which encode a total of 27,597 amino acids. The most frequently used amino acids were Leu (10.59%), followed by Ile (8.39%), Ser (7.48%), Gly (6.71%), and Arg (6.36%), respectively. The tRNA genes of *P. cuspidatum* vary between 48 and 88 nucleotides, with the GC content varying between 41.10% and 62.16%. Moreover, a total of 66 small single repeats are identified in the cp of *P. cuspidatum*, ranging from 10 bp to 16 bp.

To further investigate the taxonomic status and phylogenetic relationship of the subfamily Polygonoideae, a phylogenetic evaluation of *Polygonum cuspidatum* and its closely associated species from the related tribes (*Polygoneae*, *Rumiceae*, *Fagopyreae*, *Persicarieae* and *Calligoneae*) was carried out based on complete chloroplast sequence. The phylogenetic tree results indicated that *P. cuspidatum* was positioned in a fully supported clade with *Fallopia sachalinensis*, suggesting a close genetic relationship between the genera of *Polygonum* and *Fallopia* (Figure 1). The results also confirmed genetic diversities between *P. cuspidatum* and its morphologically similar species *Fallopia multiflora* and *Rheum palmatum,* indicating the potential of developing efficient molecular markers from cp genome (Figure 1). Since the extensive ecological and morphological variations, at least five haplotypes have been identified in *P. cuspidatum* from different regions inferred by chloroplast gene *rbcL* and *accD* (Inamura et al. 2000). The multiple alignment of *rbcL-accD* gene among *P. cuspidatum* of Zhejiang Province and other haplotypes deposited in GenBank identified a total of eleven mutation sites and indicated a unique haplotype of *P. cuspidatum.* The complete chloroplast genome of *P. cuspidatum* in this study would be valuable genetic resources for studying the population genetics, taxonomy and evolutionary relationships of Polygonoideae species.

**Figure 1.**
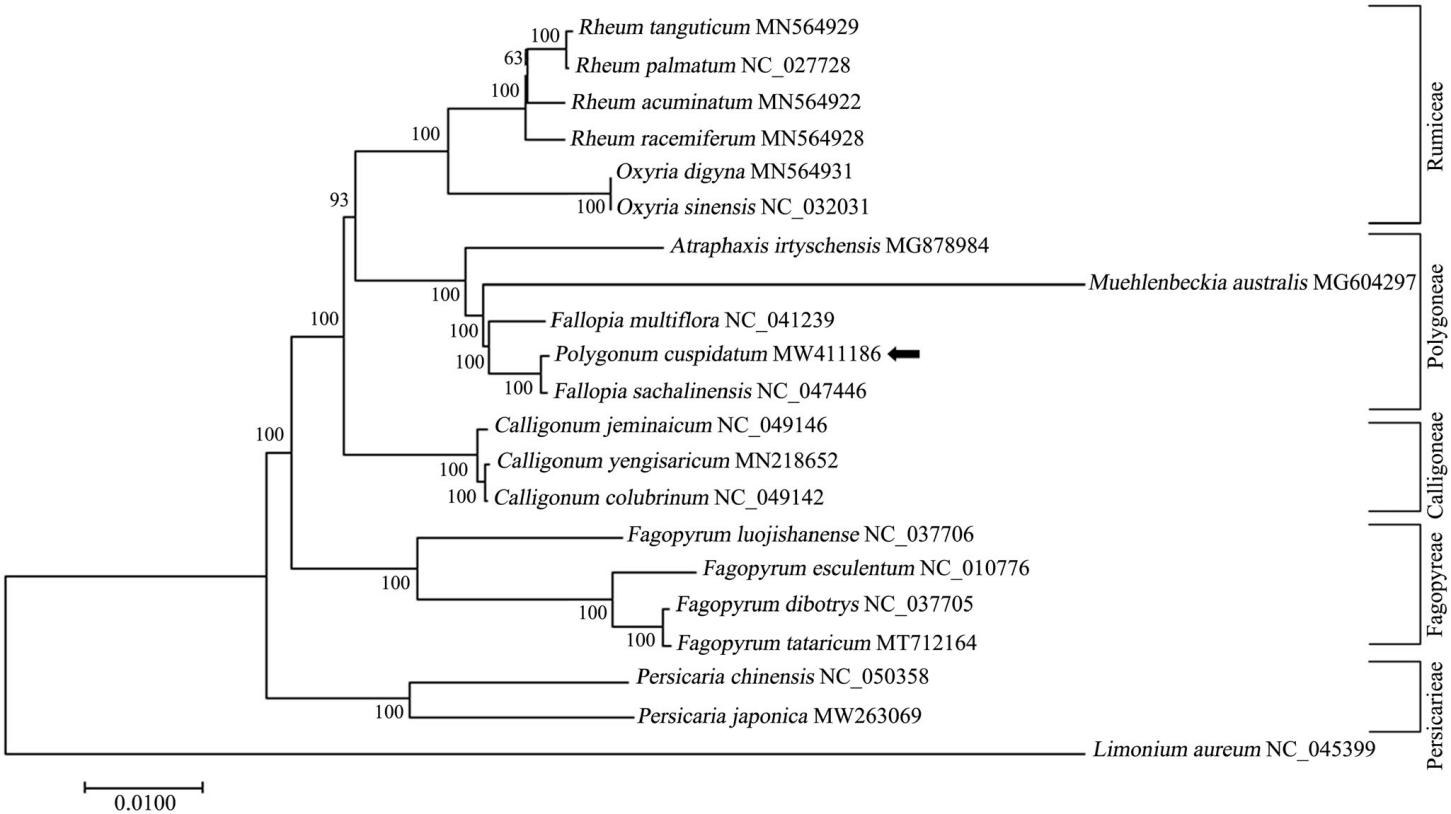
Phylogenetic relationships of the newly sequenced *Polygonum cuspidatum* and other representative species from the subfamily Polygonoideae based on complete chloroplast genomes. The tree was generated by MEGA 7.0 with maximum-likelihood (ML) method with models of K2P and G + I. The species of *Limonium aureum* from genus *Limonium* of family Plumbaginaceae served as the out-group. The newly determined chloroplast genome of *P. cuspidatum* is indicated with a black arrow. Numbers on the nodes are bootstrap values from 100 replicates. The GenBank accession numbers were listed following the species name.

## Data Availability

The genome sequence data that support the findings of this study are openly available in GenBank of NCBI at (https://www.ncbi.nlm.nih.gov/) under the accession no. MW411186. The associated BioProject, SRA, and BioSample numbers of Polygonum cuspidatum are PRJNA700824, SRR13664599 and SAMN17839755, respectively.
